# *Phaeoacremonium tuscanicum* and *Phaeoacremonium indicum* sp. nov. associated with subcutaneous phaeohyphomycosis

**DOI:** 10.1080/21501203.2024.2312917

**Published:** 2024-02-12

**Authors:** Lizel Mostert, Margaret Whipp, Alan Woodgyer, Richard C. Summerbell, David Gramaje, Chris F.J. Spies

**Affiliations:** a Department of Plant Pathology, University of Stellenbosch, Stellenbosch, South Africa; b Microbiological Diagnostic Unit Public Health Laboratory, Department of Microbiology and Immunology, The University of Melbourne at the Peter Doherty Institute of Infection and Immunity, Victoria, Australia; c Sporometrics, Toronto, Canada; d Dalla Lana School of Public Health, University of Toronto, Toronto, Canada; e Instituto de Ciencias de la Vid y del Vino (ICVV), Consejo Superior de Investigaciones Científicas - Universidad de la Rioja - Gobierno de La Rioja, Logroño, Spain; f Agricultural Research Council – Plant Health and Protection, Stellenbosch, South Africa

**Keywords:** Phaeohyphomycosis, *Phaeoacremonium*, beta-tubulin, actin

## Abstract

Two cases of phaeohyphomycotic infections were caused by *Phaeoacremonium tuscanicum*, not previously identified in human infections, and one new species, *Phaeoacremonium indicum*, respectively. Morphological and cultural investigation as well as phylogenetic analysis was constructed based on maximum likelihood analyses using actin and -tubulin sequences to identify the fungal isolates.

## 1. Introduction

The genus *Phaeoacremonium* (family Togniniaceae) currently consists of 67 species, more known for its occurrence on trees and vines, being causal agents of die-back and decline symptoms (Crous et al. 1996; Spies et al. 2018; Shang et al. 2021; Phukhamsakda et al. 2022). However, 11 species of *Phaeoacremonium* have been reported as causing human infections with *P. parasiticum* and *P. krajdenii* being the predominant species (Colombier et al. 2015; Gramaje et al. 2015). Infection types yielding *Phaeoacremonium* species include phaeohyphomycosis, mycetoma, and eumycetoma, in all cases usually caused by a traumatic inoculation (Mostert et al. 2006; Gramaje et al. 2015). *Phaeoacremonium* species identification based only on morphological and cultural characteristics can be difficult because of the overlap among different species. Using sufficiently species-discriminatory barcoding genes (β-tubulin and actin) for identification has become the standard for *Phaeoacremonium* isolates from human or plant infections (Colombier et al. 2015; Spies et al. 2018). In the present study, *Phaeoacremonium* isolates were obtained from two cases of subcutaneous phaeohyphomycosis, one from India and one from Australia. Morphological and cultural characteristics were combined with phylogenetic analysis of actin and β-tubulin gene sequences for the identification of the *Phaeoacremonium* isolates obtained from these cases.

## 2. Case reports

### 2.1. Case presentation 1

A 42-year-old male patient from India underwent a renal transplantation in 1997. The post-transplant course was uneventful and he was treated with a maintenance course of immunosuppressive therapy. In 2004, he stopped his medications and his creatinine started to increase. Then he presented himself in the hospital with anaemia and signs of acute rejection. His immunosuppressive medication was restarted. The patient noticed a small swelling on his right knee which had been present over four years. The swelling increased in size and became painful and uncomfortable, especially during walking. The patient did not remember any trauma to the knee. He was referred to the surgeon who aspirated pus from the swelling and surgically excised the entire lesion. The surgical findings showed an infected bursa. The excised tissue and pus were sent for laboratory and histopathological examination. Histopathological diagnosis confirmed a fungal abscess. The pus from the lesion was cultured on Sabouraud dextrose agar with chloramphenicol (Sab+C) and Sab+C containing cycloheximide. After 7 to 10 days of incubation at 25 °C, greyish brown to dark grey colonies were evident on both media. After the surgical excision of the lesion, his wound healed without any sequelae. He was treated with itraconazole and after one year, the patient was lost to follow-up. The culture was deposited in the Plant Pathology Stellenbosch University culture collection (STE-U 6377).

### 2.2. Case presentation 2

The patient was a 53-year-old male, living in suburban Melbourne with an infected olecranon bursa (right elbow) in November 2006. The lesion was symptomatic. Tissue, skin, and bursa fluid were surgically removed and sent for histological investigation and cultured for possible bacteria and fungi. The bacterial culture showed no bacteria; however, the fungus did grow. Fungal cultures from tissue, skin over the bursa, and the bursa fluid all grew a pure culture of a Phaeoacremonium-like fungus. The histopathologic report showed florid active chronic and granulomatous bursitis. No foreign material crystals or infectious organisms were identified and no further specimens were received from this patient. The lesion was surgically removed and there has been no further follow-up. The culture was deposited in the Plant Pathology Stellenbosch University culture collection (STE-U 6386).

## 3. Microscopic examination and culture descriptions

Strains were plated onto malt extract agar (MEA; 2% malt extract, Oxoid Ltd., Hampshire, UK; 1.5% agar, Difco, Franklin Lakes, NJ, USA) and incubated at 25 °C in the dark for 2–3 weeks. Microscopic examination was made as described by Mostert et al. (2006) and Spies et al. (2018). A mycelial plug (5 mm diam.) from the growing edge of a mycelial colony was placed in the middle of a Petri dish containing MEA, PDA, and oatmeal agar (OA). Plates were incubated at 25 °C and the colony characters and pigment production (Rayner 1970) were noted after 8 and 16 d. Cardinal temperatures for growth were determined by incubating MEA plates in the dark at temperatures ranging from 5 to 40 °C in 5 °C intervals and radial growth was measured after 8 d at 25 °C.

## 4. DNA isolation, amplification, and analysis

Genomic DNA was extracted from two strains using approximately 200 mg mycelium with the FastDNA Kit (Bio101, Carlsbad, CA, USA) according to the manufacturer’s instructions. The partial β-tubulin (*TUB2*) and actin (*ACT*) genes were amplified using the primers, T1 (O’Donnell and Cigelnik 1997) and Bt2b (Glass and Donaldson 1995), and ACT-512F and ACT-783 R (Carbone and Kohn 1999), respectively. The PCRs were done according to Mostert et al. (2006) and PCR products were sequenced as described by Spies et al. (2018). Sequences were deposited in GenBank (STE-U 6386, *ACT*-OR553104, *TUB2*-OR553105; STE-U 6377, *ACT*-OR553103, *TUB2*-OR553106). The sequences were aligned with related reference sequences of known *Phaeoacremonium* species available on GenBank using MAFFT sequence alignment program version 7. Alignments were inspected and trimmed in Geneious 9.1.8. The best-fit substitution models for each alignment were estimated under the Akaike information criterion using jModeltest 2. The *TUB2* and *ACT* alignments were concatenated to make it possible to perform a combined analysis. Maximum likelihood analyses of the concatenated alignment were conducted under the HKY+I+G model (best-fit model for both ACT and TUB) using PhyML version 3.3 with bootstrap support calculated from 100 bootstrap replicates. Bayesian analysis was conducted in PhyloBayes-MPI v. 1.9 under the CAT-GTR model. Two chains were run for 10,000 iterations of which the first 2,000 were discarded as burn-in before assessing convergence. The minimum effective sizes after running these commands were larger than 300 and maxdiff values were less than 0.1, indicating sufficient convergence as per the guidelines set out in the PhyloBayes-MPI manual. The alignment and phylogenies are available on TreeBASE (TreeBASE accession number: S30449).

**Figure 1.**
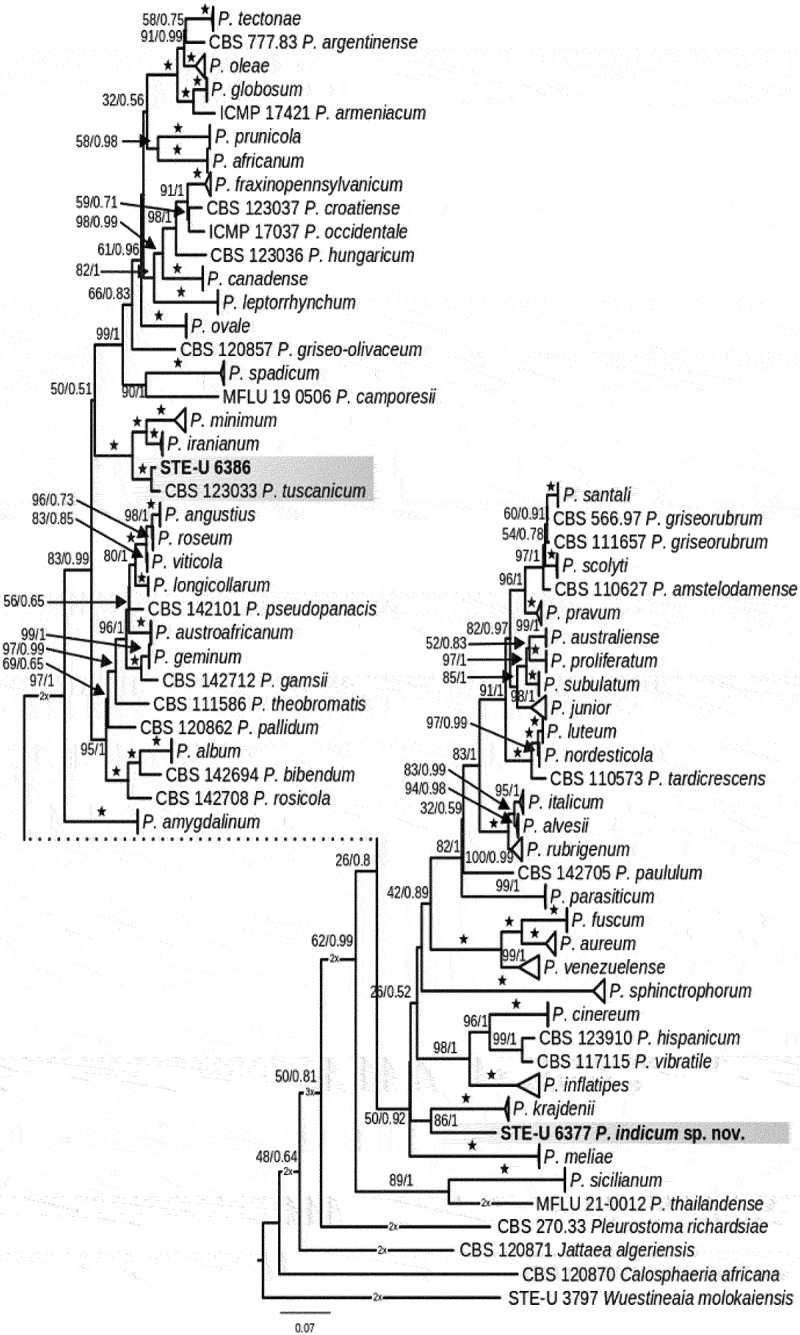
Maximum likelihood phylogeny of the genus *Phaeoacremonium* based on concatenated alignments of the β-tubulin (*TUB2*) and actin (*ACT*) regions. Bootstrap support (≥50%) and Bayesian posterior probability values (≥0.60) are indicated on the branches. Branches with complete support (100/1) are indicated with star symbols (★). Species included in this study are shaded with grey, and strains from this study are indicated in bold font. Clades with multiple strains of the same species have collapsed. *Calosphaeria africana* (CBS 120,870), *Jattaea algeriensis* (CBS 120,871), and *Wuestineaia molkaiensis* (STE-U 3797) were included as outgroup taxa.

Isolate STE-U6377 did not cluster with any of the other known species of *Phaeoacremonium* (Figure 1), however, it did group with *P. krajdenii* (bootstrap support of 86% ML and 1.00 BYPP). According to DNA sequence analyses and morphological characters, STE-U6377, could be identified as a new species.

### Phaeoacremonium indicum

**Figure 2.**
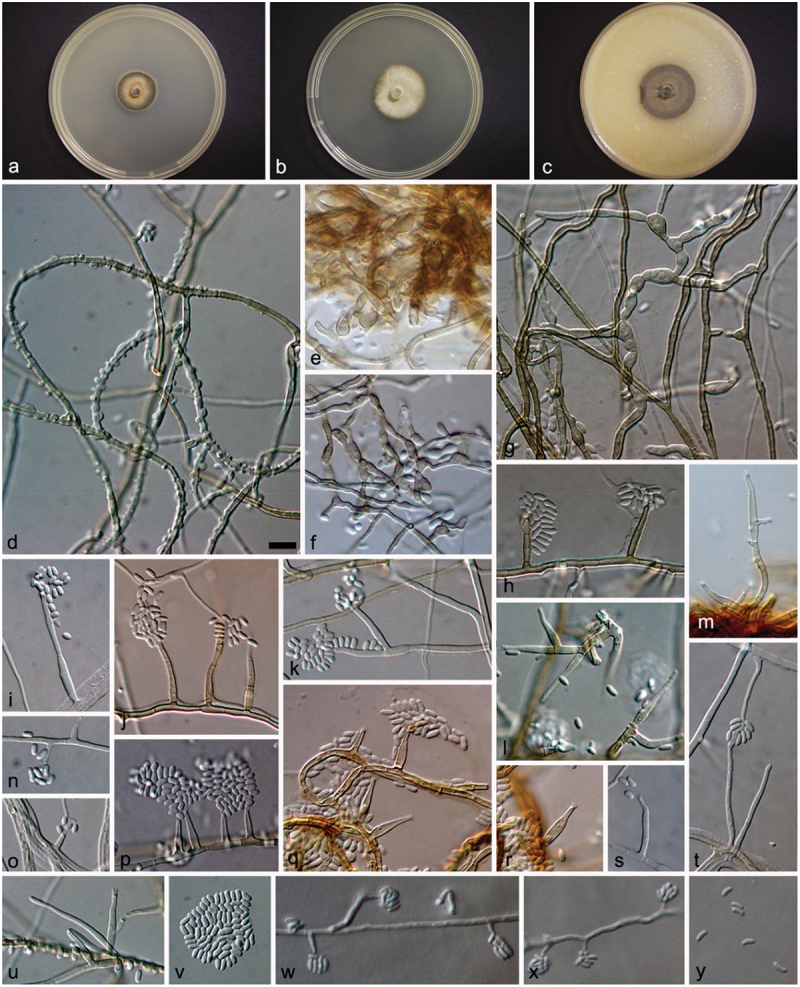
*Phaeoacremonium indicum* (STE-U 6377, ex-type). (a – c) Sixteen-day-old colonies on MEA (a), PDA (b), and OA (c). (d – v) Aerial structures on MEA; (d) Mycelium showing prominent exudate droplets observed as warts; (e – g) Mycelium with hyphal swellings; (h – j) Single conidiophores; (k – m) Branched conidiophores; (n – p) Type I phialides; (q – s) Type II phialides; (t – u) Type III phialides; (v) Conidia. (w – y) Structures on the surface of and in MEA; (w – x) Phialides and adelophialides with conidia; (y) Conidia. Scale bar: d–y = 10 µm.

Mostert L., Brandt, & Padhye sp. nov. Figure 2

MycoBank: 850314.

*Etymology:* Named after the country from which the isolate originates.

Aerial structures *in vitro* on MEA – **Mycelium** consisting of branched, septate hyphae that occur singly or in bundles of up to 13; hyphae densely tuberculate with warts up to 3 µm diam., verruculose to smooth, medium brown, 1.5–3 µm wide, hyphal swellings common, inflated cells up to 6 µm in width. **Conidiophores** are mostly short, usually unbranched, arising from aerial or submerged hyphae, erect to slightly flexuous, up to 3-septate, often ending in a single terminal phialide, medium brown to pale brown, tuberculate or verruculose on the lower part, (11–) 18.5–42.5 (−55) (av = 28.7) µm long and 1.5–3 (av = 2.2) µm wide. **Phialides** terminal, often polyphialidic, smooth to verruculose, medium brown to pale brown, collarettes, 1–2 µm long, 1–1.5 µm wide; type I phialides mostly cylindrical, 2.5–9 (−10) × 1–3 (av = 5.5 × 2) µm; type II phialides subcylindrical or navicular, 9–16 × (1.5–) 2–3 (av = 12.5 × 2.5) µm; type I and II phialides predominant; type III phialides subcylindrical, some inflated at the base, (15–) 16–24 (−26) × 1.5–3.5 (av = 19.5 × 2.5) µm. **Conidia** hyaline, mostly reniform, 4–5.5 (−6) × 1.5–2.5 (av = 4.5 × 2) µm, L/W = 2.4.

On the surface or submerged in the agar – **Phialides** hyaline, mostly cylindrical, some subcylindrical, 2.5–13 (−16) × 1.5–2.5 (−3) (av = 5.5 × 2) µm. **Conidia** hyaline, allantoid to oblong-ellipsoidal, 4–6 × 1–2 (av = 4.5 × 1.5) µm, L/W = 3.6.

*Cultural characteristics:* Colonies reaching a radius of 3–4 mm after 8 d at 25 °C. Minimum temperature for growth 15 °C, optimum 30 °C, maximum 37 °C. Colonies on MEA flat, felt, with entire margin; after 8 d, isabelline (17’’i) above, sepia (13’’k) to brown vinaceous (7’’’m) in reverse; after 16 d isabelline (17’’i) with brown vinaceous (7’’’m) undertones above, brown vinaceous (7’’’m) in reverse. Colonies on PDA flat, felty, with entire margin; after 8 d, olivaceous buff (21’’’d) above, isabelline (17’’’d) in reverse; after 16 d hazel (17’’’i) to olivaceous buff (21’’’d) above, hazel (17’’’i) in reverse. Colonies on OA flat, felty with few woolly tufts, with entire margin; after 8 d and 16 d sepia (13’’k) to greyish sepia (15’’’’i) above.

*Substrate*: Human.

*Known distribution:* India.

*Specimens examined:* INDIA, ex tissue, 42-year-old male patient who underwent renal transplantation and developed a hyphal swelling on his knee, Dr. A.A. Padhye, 2004. STE-U 6377 (holotype designated here, metabolically inactive state), P239 (culture collection of Dr. A.A. Padhye).

*Notes*: *Phaeoacremonium indicum* can be distinguished by its slow growth on MEA (3–4 mm after 8 d at 25 °C) in combination with its formation of sepia-coloured colonies on OA. Only *P. vibratile* has a similar growth rate of 3.5–4 mm after 8 d at 25 °C. *Phaeoacremonium vibratile* has buff-yellow colonies on MEA and white colonies on OA in comparison with the isabelline colonies on MEA and sepia colonies on OA of *P.* *indicum*. Distinct features of *P*. *indicum* include the medium brown mycelium, large warts, and hyphal swellings in the mycelium. *Phaeoacremonium parasiticum* also has warts of up to 3 µm, but can be distinguished by having long and branched conidiophores and predominant type III phialides. Phylogenetically *P. indicum* is closely related to *P. krajdenii*. Both these species have brown colonies on MEA and OA. *Phaeoacremonium krajdenii* has a faster growth rate (9–14 mm after 8 d at 25 °C) and smaller warts (up to 1 µm).

Isolate STE-U6386 clustered with *P. tuscanicum* with a 100% bootstrap support and 1.00 posterior probability, but differed from the type strain of that species by nine nucleotides in the β-tubulin gene and two nucleotides in the actin gene. The morphological and cultural features were similar to *P. tuscanicum*. STE-U 6386 produced yellow pigment on OA and had a maximum growth temperature of 37 °C, similar to *P. tuscanicum*. STE-U 6386 is culturally different from *P. tuscanicum* in that it does not form coremium-like structures on MEA and has olivaceous coloured colonies on MEA and PDA in comparison with the pale brown and brown colonies of *P. tuscanicum* on those media. STE-U 6386 had a colony radius of 4–4.5 mm after 8 d at 25 °C in comparison with the 8 mm for *P. tuscanicum*. Morphologically the different structures are similar in size and shape; however, the conidia of STE-U 6386 were longer and thinner than those of the ex-type, as reflected in the L/W of 3.6 for aerial conidia in comparison with 2 for *P. tuscanicum*. Both type II and III phialides were found commonly for STE-U 6386 and for *P. tuscanicum* only type II phialides were found commonly. The specimen examined came from Melbourne Australia from a 53-year-old male with an infected olecranon bursa (right elbow), isolated by Margaret Whipp in November 2007 (CBS121490, STE-U 6386, AUSMDU00085591).

## 5. Discussions

In this study, two cases of subcutaneous infections were associated with *Phaeoacremonium* species, *P. tuscanicum*, not known from humans, and one new species, *P. indicum*. Of the 11 *Phaeoacremonium* species that have been isolated from human infections, the majority have also been isolated from woody hosts (Gramaje et al. 2015; Spies et al. 2018). The first report of *P*. *tuscanicum* was from a 100-year-old vineyard showing esca symptoms in Italy (Essakhi et al. 2008). Since then, this species has been reported from oak trees, nut crops, and peach trees in Iran (Soltaninejad et al. 2017; Bashari et al. 2020; Sohrabi et al. 2020). More recently *P. tuscanicum* has also been isolated from visually healthy nursery vines in Turkey (Akgül et al. 2023).

In one of the case studies, the patient had received immunosuppressive therapy for several years, which contributed to his susceptibility to infection and disease development. In a review of 42 patients from whom *Phaeoacremonium* spp. were isolated from infections, 74% of the patients were found to be immunocompromised because of organ or bone marrow transplantation with accompanying immunosuppressive medication (Colombier et al. 2015). Patients receiving immunosuppressive therapy are more prone than immunocompetent patients to infection by these opportunistic fungal species.

In both cases in this study, no traumatic event was reported to be associated with acquiring the phaeohyphomycotic infection. It has been speculated that infections can occur due to infected wood splinters that become injected into the skin (Mostert et al. 2005), however, there are few actual reports of this. The environmental sources apart from woody hosts of *Phaeoacremonium* spp. include soil and air (Spies et al. 2018; Halleen et al. 2020). Infections due to wounds on the skin together with exposure to conidia in the soil, would be a possibility for gardeners or farmers working with hands in the soil or walking on bare feet. Cases of foot infections (Belkin et al. 2020) and finger infections support this hypothesis (Colombier et al. 2015).

In our first case, a mycetoma from the knee was removed surgically and the patient was treated with itraconazole. After one year the patient showed no further signs. In the second case, the lesion on the elbow was surgically removed and no further follow-up was noted. It would seem that the treatment of these two cases was successful. The therapies used – excision plus itraconazole in an immunocompromised patient, and wide excision alone to remove a limited lesion in an immunocompetent patient – both represent methods of therapy that are well supported in the review literature (Colombier et al. 2015).
